# Semi-Mechanistic Modeling of Florfenicol Time-Kill Curves and *in silico* Dose Fractionation for Calf Respiratory Pathogens

**DOI:** 10.3389/fmicb.2019.01237

**Published:** 2019-06-11

**Authors:** Ludovic Pelligand, Peter Lees, Pritam Kaur Sidhu, Pierre-Louis Toutain

**Affiliations:** ^1^Royal Veterinary College, Department of Comparative Biomedical Sciences, Hawkshead Campus, Hatfield, United Kingdom; ^2^Institute of Computational Comparative Medicine, College of Veterinary Medicine, Kansas State University, Manhattan, KS, United States; ^3^École Nationale Vétérinaire de Toulouse, Toulouse, France

**Keywords:** PK/PD, modeling and simulation, time-kill assay, antimicrobial susceptibility testing, *Pasteurella multocida*, *Mannheimia haemolytica*, VetCAST, bovine respiratory disease

## Abstract

An important application of time-kill curve (TKC) assays is determination of the nature of the best PK/PD index (*f*AUC/MIC or *f*T% > MIC) and its target value for predicting clinical efficacy *in vivo*. VetCAST (the veterinary subcommittee of EUCAST) herein presents semi-mechanistic TKC modeling for florfenicol, a long acting (96 h) veterinary antimicrobial drug licensed against calf pneumonia organisms (*Pasteurella multocida* and *Mannheimia haemolytica*) to support justification of its PK/PD_breakpoint_ and clinical breakpoint. Individual TKC assays were performed with 6 field strains of each pathogen (initial inoculum 10^7^ CFU/mL with sampling at times at 0, 1, 2, 4, 8, and 24 h). Semi-mechanistic modeling (Phoenix NLME) allowed precise estimation of bacteria growth system (K_GROWTH_, natural growth rate; K_DEATH_, death rate; B_MAX_, maximum possible culture size) and florfenicol pharmacodynamic parameters (E_MAX_, efficacy additive to K_DEATH_; EC_50_, potency; Gamma, sensitivity). PK/PD simulations (using the present TKC model and parameters of a florfenicol population pharmacokinetic model) predicted the time-course of bacterial counts under different exposures. Of two licensed dosage regimens, 40 mg/kg administered once was predicted to be superior to 20 mg/kg administered at 48 h intervals. Furthermore, we performed *in silico* dose fractionation with doses 0 – 80 mg/kg administered in 1, 2 or 4 administrations over 96 h and for MICs of 0.5, 1, 2, 4 mg/L with 2 inoculum sizes 10^5^ and 10^7^ CFU/mL. Regression analysis (I_max_ model) demonstrated that i) *f*AUC/MIC outperformed *f*T% > MIC as PK/PD index and ii) maximum efficacy (IC_90%_) was obtained when the average free plasma concentration over 96 h was equal to 1.2 to 1.4 times the MIC of *Pasteurella multocida* and *Mannheimia haemolytica*, respectively.

## Introduction

The aim of time–kill *in vitro* assays is to investigate the pharmacodynamics (PD) of antimicrobial drugs (AMD) by determining the rate of bacterial kill relative to drug concentration. Quantitative analysis of time-kill curve (TKC) data is more informative of the drug-bacteria relationship than snapshot indices, such as minimum inhibitory concentration (MIC). MIC indicates only the net effect of a single AMD concentration on bacterial growth over a 24 h incubation period, while TKC establishes the rate of killing over a range of concentrations. Based on TKC data AMDs can be classified as time- or concentration-dependent in killing action (Toutain et al., 2017).

An important application of TKC data is determination of the best PK/PD index (*f*AUC/MIC or *f*T > MIC) for predicting clinical efficacy *in vivo*, where *f*AUC is area under plasma concentration-time curve and *f*T is the time the drug concentration exceeds MIC, for free drug concentrations. This has historically been established by correlating the reduction in bacterial count at 24 h from an initial inoculum count (Lees et al., 2015). Plots of log10 colony forming units (CFU)/mL at 24 h versus each of the two PK/PD indices allowed selection of the PK/PD index which best fits the sigmoidal E_MAX_ model (Lees et al., 2015). This approach was based on the net reduction of bacterial count with each concentration exposure, but did not utilize the time course (i.e., the shape) of individual kill curves. For human medicine, several advanced PK/PD models of TKC have incorporated the shape of the curve with time (Nielsen et al., 2007; Nielsen and Friberg, 2013, model D, Figure 5). This more advanced modeling enables estimation of the three pharmacodynamic (PD) parameters of AMD action, namely potency, efficacy and sensitivity. This approach therefore allows characterization the whole concentration-effect relationship.

In the present investigation, using historical TKC data (Sidhu et al., 2014), PD parameters of florfenicol against the calf pneumonia organisms, *Pasteurella multocida* (*P. multocida*) and *Mannheimia haemolytica* (*M. haemolytica*), have been established using the semi-mechanistic model proposed by Nielsen and Friberg (2013). The objective was to then conduct an *in silico* dose fractionation trial to determine the PK/PD index for florfenicol and these pathogens, best correlating with bacterial kill. Dose fractionation studies are generally conducted *in vivo* using rodent infection models, whereas in this study semi-mechanistic PD florfenicol models were used as a surrogate of rodent models to predict microbiological effects in response to a range of florfenicol dosage regimens. The ultimate goal was to compute a PK/PD breakpoint (PK/PD_BP_) for the florfenicol clinical breakpoint (CBP) according to the procedures advocated by VetCAST (Toutain et al., 2017), where CBP is the MIC value used by microbiology laboratories to report the results of antimicrobial sensitivity testing (AST). PK/PD_CO_ is defined as the highest possible MIC for which a given percentage of animals in the target population (say 90%) achieve a pre-defined target value of the PK/PD index, the pharmacodynamic target (PDT) according to European Medicines Agency (EMA) terminology.

## Materials and Methods

### Test Pathogens and MIC Determination

The test pathogens were *P. multocida* and *M. haemolytica*. MICs were determined in Mueller Hinton Broth (MHB) for six strains of each species, isolated from cases of calf pneumonia (Sidhu et al., 2014). Their origin and date of isolation are summarized in the Supplementary File S1. Average florfenicol MICs were determined using 5 overlapping 2-fold-dilution series and were 0.4 and 0.5 mg/L for *P. multocida* and *M. haemolytica*, respectively.

### Time-Kill Curves

Six individual TK assays were performed for each pathogen. Initial inoculum count was in the range 5 × 10^6^ to 7 × 10^7^ CFU/mL. Duration of incubation was 24 h with sampling at times of 0, 1, 2, 4, 8, and 24 h. Drug concentrations were expressed in the initial publication (Sidhu et al., 2014) as multiples of MIC (0 = growth control, 0.25, 0.5, 1, 2, and 4 times measured MIC). For data fitting, MICs were back calculated to mg/L. The lowest detectable count was 33 CFU/mL; lower counts were set as below the quantification limit (BQL). All TKC data sets analyzed for this study are included in the manuscript and the supplementary File S2.

### Data Analysis

**FIGURE 1:**
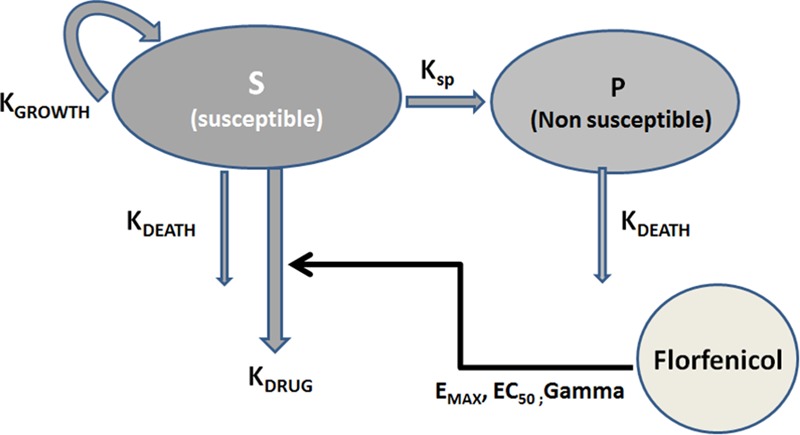
Semi-mechanistic model for Time-Kill curve modeling, described in Nielsen et al. (2007) and Nielsen and Friberg (2013). *K_DEATH_* (*per h*) *is the natural death rate for both S* (*susceptible*) *and P* (*non-susceptible*) *pools; K_GROWTH_* (*per h*) *is the growth rate of S; B_MAX_* (*CFU/mL*) *is the maximum possible size of the culture* (*S+P*). *S+P constitutes the total bacterial count. For the starting inoculum, all bacteria were assumed to be in the growing, drug-sensitive stage. As the total bacterial content in the system increases, bacteria are progressively transferred into the P resting pool. This transfer was modeled by an irreversible rate constant* (*K_SP_*) *between S and P. K_SP_ was parametrized in terms of B_MAX_, K_GROWTH_ and K_DEATH_* (*natural death rate*) *as described in* Nielsen et al. (2007) *and* Nielsen and Friberg (2013). *E_MAX_* (*1/h*) *is a maximal killing rate of bacteria for the susceptible pool* (*additional to natural death rate*), *EC_50_ is the florfenicol in vitro concentration* (*mg/L*) *for E_MAX_/2 and Gamma* (*a scalar*), *the Hill coefficient; E_MAX_, EC_50_ and Gamma are the three PD parameters allowing quantification of florfenicol efficacy, potency and sensitivity, respectively.*

Pharmacodynamic data analyses were conducted using Phoenix^®^ WinNonlin^®^ 8.0 (Pharsight Corporation, St Louis, MO, United States). For each pathogen, the 6 TKC data sets were analyzed simultaneously using a non-linear mixed effect model (NLME). A semi-mechanistic structural model of bacterial growth, incorporating a compartment for growing drug-sensitive bacteria (S) (CFU/mL) and a compartment named persisters (P) (CFU/mL), corresponding to a pool of non-growing and insensitive-drug bacteria (phenotypic resistance) was adopted (Figure 1; Nielsen and Friberg, 2013).

Visual Inspection of TKC indicates an initial phase of slow growth. To capture the delay required to achieve a maximal steady-state growing rate, a mitigating function for *K_GROWTH_* of the form was introduced (Equation 1):

(1) $$K_{GROWTH}=K_{GROWTHMAX}\times \left(1-EXP\left(-Alpha\times Time\right)\right)$$

where Alpha (per h) = rate constant to describe a progressive increase of *K_GROWTH_* over time; at time 0, *K_GROWTH_* = 0 then, *K_GROWTH_* increases progressively to reach *K_GROWTHMAX_* with a mean time equal to *1/Alpha*. The lag phase corresponds to the physiological adaptation of the bacteria to the culture condition (induction of specific messenger RNA and protein synthesis and low cell density accounting for initial dilution of the exoenzymes that make nutrients readily available).

Florfenicol action was introduced in the model as a concentration-dependent killing rate *K_DRUG_*_(_*_t_*_)_ (per h) acting in parallel with *K_DEATH_* but for the S pool only. It was modeled according to the classical Hill equation (Eq. 2).

(2) $$K_{DRUG\left(t\right)}=\frac{E_{MAX}\times C{\left(t\right)}^{Gamma}}{EC_{50}^{Gamma}+C{\left(t\right)}^{Gamma}}$$

where *C*(*t*) is the florfenicol concentration (mg/L) at time *t* (the independent variable). *C*(*t*) was the constant tested concentration when data were fitted to estimate the PD parameters but *C*(*t*) was obtained by solving the population PK model, when this equation was used for simulations (*vide infra*). *E_MAX_* (1/h) is the maximal killing rate for the susceptible pool (additionally to natural death rate), *EC_50_* is the florfenicol *in vitro* concentration (mg/L) for E_MAX_/2 and *Gamma* (a scalar), the Hill coefficient; *E_MAX_, EC_50_* and *Gamma* are the three PD parameters providing quantitative indices of florfenicol efficacy, potency and sensitivity, respectively.

There were substantial differences in *B_MAX_* across the six field strains of each pathogen. Therefore, a random component was introduced in the structural model to account for the inter-strain variability. Six individual *B*_MAX_ values were obtained, using an exponential model of the form (Eq. 3):

(3) $$\theta_{1i}=\;\theta_{1}\times Exp\left(\eta_{1i}\right)$$

where 𝜃_1_ is the typical population value of *B_MAX_*, 𝜃_1i_ the value of *B_MAX_* for the *i^th^* TKC assay, and η_1i_ (*eta*) the deviation associated with the *i^th^* strain from the corresponding population value. This exponential model assumes a log-normal distribution of *B_MAX_*. The between-strain variability of *B_MAX_* was reported as coefficient of variation in the original scale, with an equation converting estimated variance terms to a coefficient of variation (CV%) (Eq. 4).

(4) $$CV\left(\%\right)=100\times \sqrt{\exp\left(\omega^{2}\right)-1}$$

The residual variability was modeled with an exponential error model of the form (Eq. 5):

(5) $$Y_{ij}=\hat{Y_{ij}}\times EXP(\varepsilon_{ij})$$

where 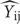 is the *j^th^* response (CFU/mL) measured in the *i^th^* curve in terms of CFU (no log-transformation of raw data), with 𝜀_ij_ the common errors term having a mean of 0 and a variance $\sigma_{1}^{2}$.

When there is only one exponential error model, the predictions and observations are automatically log-transformed by Phoenix and fitted in that space, so that the error model was actually a Log-additive error model.

Parameter estimates, with their associated SE and CV as a measure of precision, were based on minimizing an objective function value, using Laplace engine for the Maximum Likelihood Estimation.

For *P. multocida*, there were no values reported as below the quantification limit (BQL) due to some re-growth at 24 h. For *M. haemolytica*, data reported as BQL (7% of the data set) were retained in the analysis by using a likelihood-based approach according to the M3 method (Beal, 2001). Diagnostic plots determined whether the model was adequate: these included PRED (population (zero-eta) prediction) and IPRED (individual prediction) versus the dependent variable, Conditional weighted residuals (CWRES) and individual fitting. The overall adequacy of the model was established by plotting the Visual Predictive Check (VPC) i.e., a graphical comparison between the observed data and prediction intervals (20–80th percentiles) derived from the simulated data (data set simulated 500 times).

Secondary parameters computed were MIC and minimal bactericidal concentration (MBC). MIC and MBC indicate AMD PD parameters (efficacy, potency, sensitivity) but also test tube conditions [growth and death rates, duration of observation (often 18–24 h) and the initial inoculum load (usually 5 × 10^5^ CFU/mL)]. According to Mouton and Vinks (2005), MIC is related to the aforementioned factors by eqs. 6A,B:

(6A) $$MIC=EC_{50}\times {\left(\frac{K_{GROWTH}-0.29}{E_{MAX}-\left(K_{GROWTH}-0.29\right)}\right)}^{\frac{1}{Gamma}}$$

where *K_GROWTH_*, (actually *K_GROWTH_*-*K_DEATH_*) (for present data it is *K_GROWTHMAX_*), *EC_50,_ E_MAX_*, and *Gamma* as defined above*;*

Time of measurement was fixed at 18 h and it is assumed that visible growth indicates an inoculum of 1 × 10^8^ CFU/mL; hence, the constant 0.29 of eq. 6A is obtained from eq. 6B:

(6B) $$\frac{1}{Time\;of\;measurement\left(18h\right)}\times LN\left(\frac{N\left(t\right)}{N\left(0\right)}\right)=0.294$$

where *N*(t) is the inoculum size at 18 h i.e., 10^8^ CFU/mL and *N*(0) is the initial inoculum i.e., 5 × 10^5^ CFU/mL. When the initial load is not 5 × 10^5^ CFU/mL as for the Sidhu et al. (2014), data eq. 6B should be edited to replace 0.29 by the *ad hoc* value; for example, using an initial count of 10^7^ CFU/mL, the constant is no longer 0.29 but -0.127.

Similarly, MBC is computed by replacing, in the previous equation, 10^8^ by 5 × 10^2^ CFU/mL; MBC corresponds to at least 99.9% kill from the initial inoculum (5 × 10^5^ CFU/mL) (Mouton and Vinks, 2005); MBC is given by eq. 7:

(7) $$MBC=EC_{50}\times {\left(\frac{K_{GROWTH}+0.383}{E_{MAX}\;-\;K_{GROWTH}+0.383}\right)}^{\frac{1}{Gamma}}$$

The Phoenix model code is available on request and will be made available by the authors, without undue reservation, to any qualified researcher.

The estimated fixed parameters (E_MAX_, EC_50_, Gamma, K_GROWTHMAX_, K_DEATH_, B_MAX_) and Alpha were reported as typical values with coefficient of variation.

### Simulation of Two Possible Dosing Regimens and *in silico* Dose Fractionation to Select a PK/PD Index for Florfenicol

Selection of the best PK/PD index for florfenicol and its magnitude were calculated using the *in silico* PK/PD model, simulating several dosage regimens using eq. 2 with *C*(*t*) being the predicted plasma florfenicol concentration obtainable *in vivo*. *C*(*t*) was determined by solving the population PK model developed for florfenicol in calves by Toutain et al. (2017), which is a meta-analysis of PK studies in which calves were administered 40 mg/kg of florfenicol subcutaneously (300 mg/mL Solution for Injection). The design of the population pharmacokinetic study and the resulting estimated PK parameters used for these simulations are presented in Supplementary File S3. Using the PK/PD *in silico* model, the microbiological effect of two possible licensed dosing regimens were simulated: single dose (40 mg/kg) versus 20 mg/kg twice at 48 h dosing interval. Using the same PK/PD model, dose fractionation was conducted for doses of 0, 2.5, 5, 10, 20, 30, 40 (licensed dose), 50, 60, and 80 mg/kg given, as a single administration, two administrations at 48 h interval or 4 administrations at 24 h intervals, yielding a total of 28 possible exposure patterns. Simulations were performed for two initial loads (10^5^ and 10^7^ CFU/mL) and for four MIC levels (0.5, 1, 2, and 4 mg/L). We assumed that differences in MIC were due solely to altered potency and not efficacy. For simulation at MICs of 0.5, 1, 2, and 4 mg/L, the EC_50_ fitted from TKCs was multiplied by a scaling factor converting measured MIC (0.4 mg/L for *P. multocida* and 0.5 mg/L for *M. haemolytica*) to the simulated MIC. For the bacteriological response, the cumulative Area Under the Curve of the total bacterial count over 96 h (AUC_bact96 h_) was used. Data were then log10 transformed for regression. When the bacterial count had decreased to 30 CFU/mL, it was considered that re-growth would not occur and curves were truncated for this cut-off value. PK/PD indices are conventionally determined using plasma protein unbound (free) concentration. The latest study at the time of writing reported that florfenicol protein binding was only 5% at the high concentration and was negligible at the low concentrations, representing a *fu* of essentially 1.0 (Foster et al., 2016). We therefore hypothesized that the binding of florfenicol to plasma protein could be ignored and that we simulated free plasma concentrations in the dose fractionation (*vide infra*). The area under the plasma concentration-time curve (*f*AUC_PK(0-96 h)_) and percentage time plasma concentration exceeded MIC within 96 h (*f*T > MIC%) were computed using the statistical tool of Phoenix. The 28 pairs of *f*T > MIC% (independent variable) versus AUC_bact96 h_ (dependent variable) and *f*AUC_PK(0-96 h)_/MIC (independent variable) versus AUC_bact96 h_ (dependent variable) obtained for each MIC were fitted with an Inhibitory Effect Sigmoid I_max_ PD model (Model 108), Eq. 8:

(8) $$Effect=E_{0}-\left(\frac{I_{max}\times INDEX^{Slope}}{INDEX^{Slope}+INDEX_{50}^{Slope}}\right)$$

where E_0_ is the maximum effect (obtained for the control curve for *C*(*t*) = 0), the maximum possible observed effect is (*E_0_-I_max_*), *I_max_* being the amplitude of maximal effect. *INDEX_50_* is the magnitude of the index (*fAUC*_PK(0-96 h)_*/MIC* or *fT > MIC%*) that achieves 50% of the *I_max_*, and *Slope* is the sigmoidicity factor, reflecting the steepness of the relationship. Curve fitting was performed with WinNonlin^®^ using the non-linear least-squares algorithm. The coefficients of determination (*R*^2^), the Akaike Information Criterion (AIC), and visual inspection of graphs were used to select the PK/PD index that best predicted the antibacterial effect. The *INDEX_90%_* was computed as the breakpoint value of the predicting PK/PD index.

## Results

### Time-Kill Curve Modeling

Parameter estimates from the TKC model (bacteria growth system and drug sub-models) are summarized in Table 1. The precision of the estimation of the parameter value was good in all cases (estimated CV% less than 38%).

**Table 1:** Primary and secondary parameter estimates and precision (CV%).

		*Pasteurella multocida*		*Mannheimia haemolytica*	
Primary parameters^a^	Unit	Estimate	CV%	Estimate	CV%
**Bacteria growth system parameters**					
K_GROWTHMAX_	1/h	0.97	5.1	1.58	10.7
K_DEATH_	1/h	0.12	11.9	0.78	11.1
B_MAX_	CFU/mL	5.2 × 10^9^	38.1	9.6 × 10^8^	34.6
alpha	1/h	0.22	8.9	0.93	12.8
**Florfenicol pharmacodynamic parameters**					
EC_50_	mg/L	0.46	7.5	0.70	7.2
gamma	scalar	2.74	8.9	2.63	11.9
E_MAX_ (maximal killing rate)	1/h	2.00	2.4	2.70	8.4
stdev0 (Ln domain)	scalar	1.11 (156 CV%)	9.9	1.05 (141 CV%)	5.5
**Secondary parameters^b^**					
MIC (10^5^ CFU/mL inoculum)	mg/L	0.35	5.9	0.43	8.1
MIC (10^7^ CFU/mL inoculum)	mg/L	0.40	6.4	0.49	7.1
stationary concentration	mg/L	0.41	6.5	0.50	7.0
MBC	mg/L	0.51	7.5	0.60	6.3

*^*a*^K_*GROWTHMAX*_, maximal growth rate; K_*DEATH*_, natural death rate; Alpha, delay required to achieve maximal steady-state growth rate; B_*MAX*_, maximum possible bacterial density; E_*MAX*_, maximal increase in killing rate in addition to K_*DEATH*_, EC_*50*_ concentration required to achieve half of E_*MAX*_; gamma, Hill coefficient. ^*b*^Minimum Inhibitory* (*MIC*) *and Bactericidal* (*MBC*) *concentrations and stationary concentrations computed according to Mouton and Vinks (6).*

Maximal growth rate (K_GROWTHMAX_, per hour) was 0.97 for *P. multocida* (yielding a 0.71 h generation half-life) and 1.58 for *M. haemolytica* (yielding a 0.44 h generation half-life). The natural death rate (K_DEATH_, per hour) was 0.12 for *P. multocida* (yielding a 5.9 h count-halving half-life) and 0.78 for *M. haemolytica* (yielding a 0.9 h count-halving half-life). The delay in achieving a maximal steady-state growing rate (Alpha) was 0.22 h^-1^ for *P. multocida* and 0.93 h^-1^ for *M. haemolytica*, corresponding to half-lives to establish full growth capacity of 3 and 0.75 h, respectively. The maximum possible bacterial density of the cultures (B_MAX_) was 5.2 × 10^9^ CFU/mL for *P. multocida* and 9.6 × 10^8^ CFU/mL for *M. haemolytica*. The CV% for inter-strain (assay) variability was 89% for *P. multocida* and 430% for *M. haemolytica*. Low values of eta-shrinkage (12% *P. multocida* and 4% for *M. haemolytica*) confirm the identifiability of the random effect on B_max_.

The maximal drug-induced increase in bacterial killing rate (E_max_, per hour) was 2.0h^-1^ for *P. multocida* (yielding a 16.7-fold increase in overall death rate) and 2.7 h^-1^ for *M. haemolytica* (yielding a 3.5-fold increase in overall death rate). The *in vitro* concentration for achieving half the maximal effect (EC_50_) was 0.46 mg/L for *P. multocida* and 0.70 mg/L for *M. haemolytica*, ranking favorably for average experimental MICs of 0.4 mg/L for *P. multocida* and 0.5 mg/L for *M. haemolytica*. The slope of the concentration-effect curve (gamma, dimensionless scalar) was for 2.74 for *P. multocida* and 2.63 for *M. haemolytica*.

**FIGURE 2:**
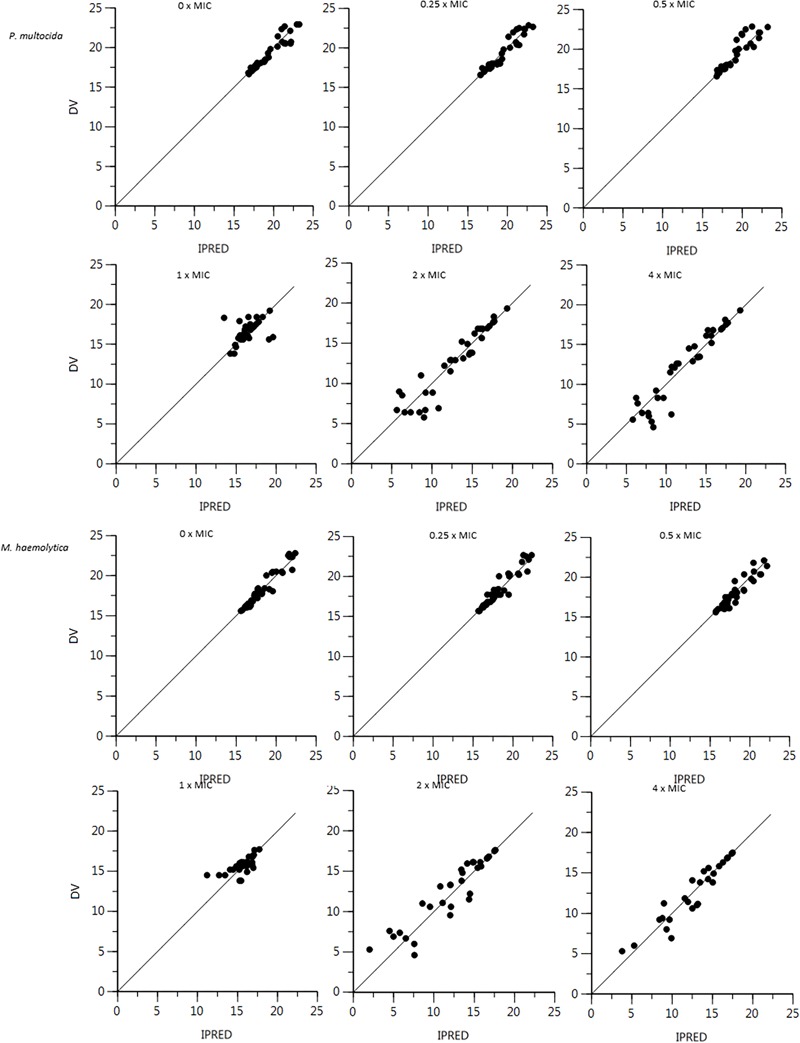
Plot of the dependent variable (DV) i.e., observed natural logarithm of bacterial counts (CFU/mL) versus individual predicted count values (IPRED) for *P. multocida* and *M. haemolytica*. *Individual strain predictions are obtained by setting random effects to the “post hoc” or empirical Bayesian estimate of the random effects for the individual from which the DV observation was made. Thus, the plot shows observed versus fitted values of the model function. Ideally, they should fall close to the line of unity y = x.*

**FIGURE 3:**
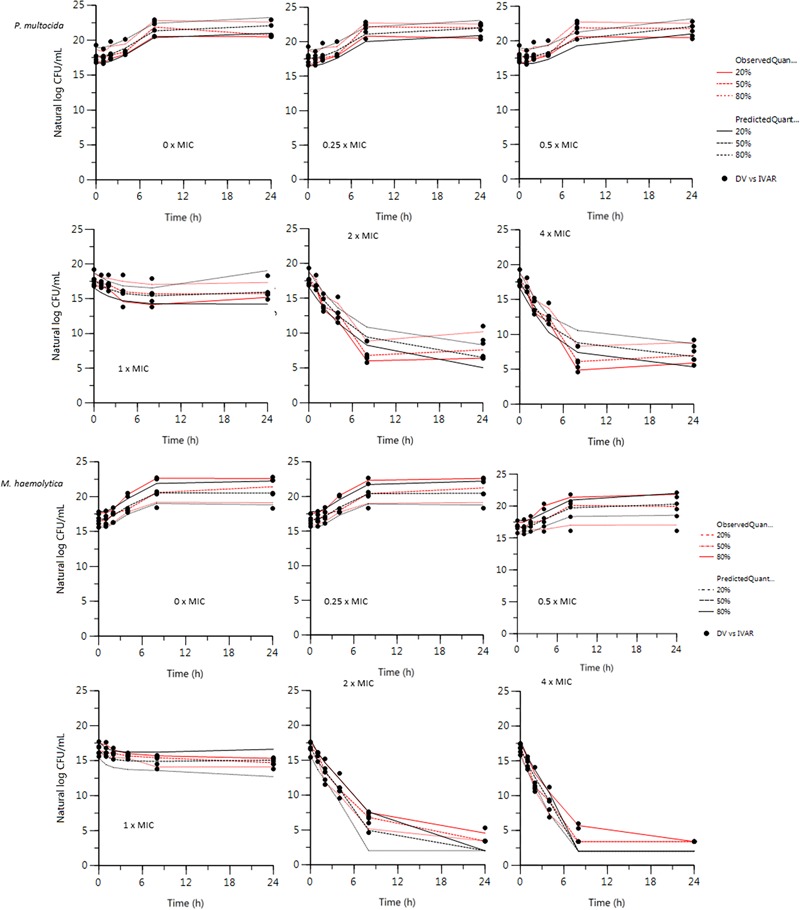
Visual Predictive Check (VPC) for *P. multocida* and *M. haemolytica*. *VPCs were obtained with 100 replicates of each set of 6 strains* (*100 × 6 × 6 = 3600 individual curves*). *For each stratification, the observed quantiles* (*20, 50, and 80%*) *are well super-imposed with the corresponding predictive check quantiles over the observed data. Theoretically, approximately 40% of data should be outside the plotted quantiles. Red lines: observed quantiles; Black lines: predicted quantiles; Black symbols: observed data.*

The plot of the observed natural logarithm of bacterial counts (CFU/mL, the dependent variable DV) versus individual predicted count values (IPRED) for *P. multocida* and *M. haemolytica* is presented in Figure 2. Visual predictive check (VPC) for *P. multocida* and *M. haemolytica* are shown in Figure 3.

### Comparison of Microbiological Response for Two Possible Modalities of Florfenicol Administration

**FIGURE 4:**
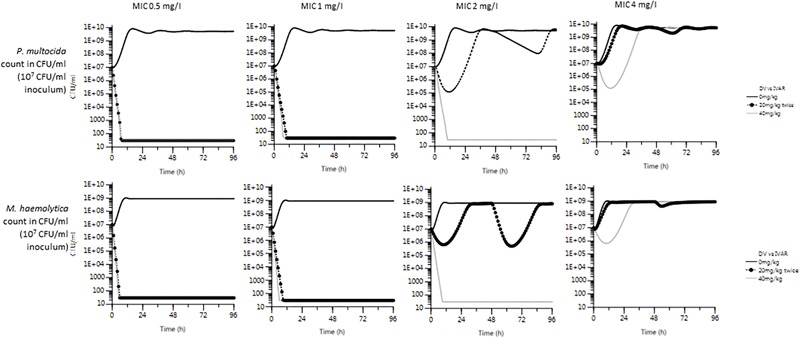
Prediction of total bacterial count of *P. multocida* and *M. haemolytica* over 96 h, for each MIC (0.5, 1, 2, and 4 mg/L), with two licensed dosage regimens: single administration of 40 mg/kg (gray line) vs. 2 doses of 20 mg/kg at 48 h interval (black line with dots). *Simulations were run for two starting inoculum counts* (*low 10^5^ and high 10^7^ CFU/mL, corresponding to metaphylactic and treatment circumstances*), *but only simulations with 10^7^ CFU/mL are illustrated. Bacterial counts were limited to 30 CFU/mL* (*thus assuming cure was achieved*).

The *in silico* predicted microbiological efficacy of the two approved dosage regimens for florfenicol were similar for two inoculum sizes (low 10^5^ and high 10^7^ CFU/mL) for *P. multocida* and *M. haemolytica* at MICs of 0.5, 1, 2, and 4 mg/L (Figure 4). For an MIC of 2 mg/L, the single administration of 40 mg/kg was clearly superior to the two administrations of 20 mg/kg at 48 h interval for both *P. multocida* and *M. haemolytica* and with both inoculum counts. For an MIC of 4 mg/L, none of the dosage regimens were predicted to be efficacious by the *in silico* PK/PD model.

### Dose Fractionation *in silico*

**FIGURE 5:**
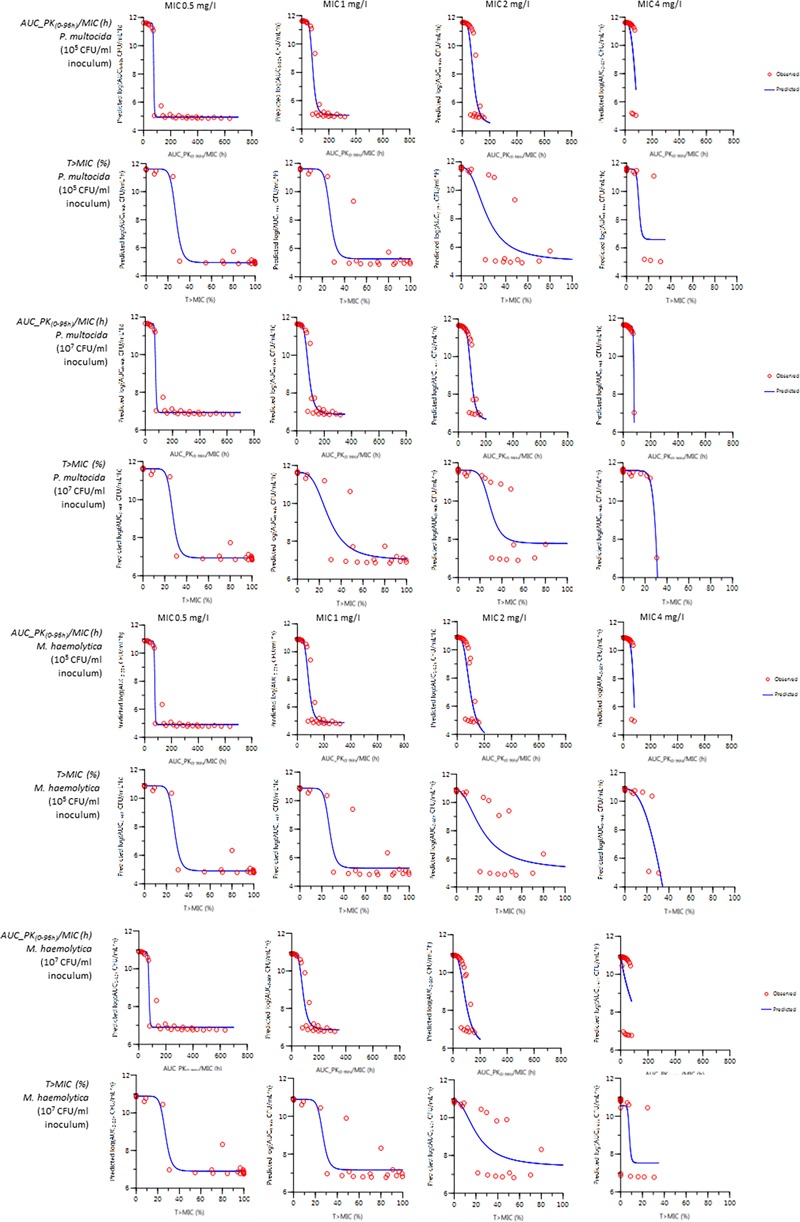
Comparison of fitting for prediction of log10 AUCbact_(0-96 h)_ (I_max_ sigmoid model) with *f*AUC_PK_(0-96 h)_/MIC or *f*T > MIC% as a predictive variable for MIC of 0.5 to 4 mg/L (from left to right) for inoculum sizes of 10^5^ and 10^7^ CFU/mL for both *P. multocida* and *M. haemolytica. A sigmoid I_max_ model predicted log10 AUCbact*_(_*_0-96 h_*_)_ *from the value of the PK/PD indices: either fAUC_PK*_(_*_0-96 h_*_)_*/MIC* (*top row*) *or fT > MIC%* (*bottom row*) *were used as a predictive variables for MICs of 0.5 to 4 mg/L* (*from left to right*) *for inoculum sizes of 10^5^ and 10^7^ CFU/mL for both P. multocida and M. haemolytica.*

Figure 5 illustrates the fitting comparison for prediction of log10AUCbact_(_0-96 h_)_ (I_max_ sigmoid model) using *f*AUC_(PK0-96 h)_/MIC or *f*T > MIC% as the predictive variable for MICs 0.5, 1, 2, and 4 mg/L and for inoculum strengths of 10^5^ and 10^7^ CFU/mL for both *P. multocida* and *M. haemolytica*. The fitting for MIC 4 mg/L was excluded due to the limited efficacy of even the highest dosage regimen. In all cases, *f*AUC/MIC was a better PK/PD index than *f*T > MIC over 96 h. For *P. multocida*, the goodness of fit values, averaged for inoculum sizes of 10^5^ and 10^7^ CFU/mL and for all MICs, were better for *f*AUC_PK(0-96 h)_/MIC (AIC = 76.9, *R*^2^ = 0.939) than for *f*T > MIC (AIC = 81.3, *R*^2^ = 0.934). For *M. haemolytica*, the goodness of fit values, averaged for inoculum sizes of 10^5^ and 10^7^ CFU/mL and for all MICs, were also better for *f*AUC_PK(0-96 h)_/MIC (AIC = 84, *R*^2^ = 0.924) than for *f*T > MIC (AIC = 86.3, *R*^2^ = 0.924).

The critical value for 90% of the maximal *in silico* possible anti-bacterial action for *f*AUC_PK(0-96 h)_/MIC was solved using Eq. 8 for the two inoculum strengths actually tested (10^5^ and 10^7^ CFU/mL) and for MICs of 0.5, 1, and 2 mg/L (Table 2).

**Table 2:** Critical value of the PK/PD index (*f*AUC_PK(0-96 h)_/MIC, unit h) to achieve 50% or 90% of the maximal possible *in silico* bacteriological effect.

Efficacy (% Emax)		*P. multocida*		*M. haemolytica*		Average free plasma concentration required over 96 h (expressed in MIC-fold, unitless) to achieve 90% of the maximal efficacy^b^	
		50%	90%	50%	90%		
	**MIC (mg/L)**	**fAUC_PK(0-96 h)_/MIC (h)^a^**				***P. multocida***	***M. haemolytica***

10^5^ CFU/mL inoculum	0.5	75	81	75	81	0.84	0.84
	1	81	**115**	83	**127**	**1.19**	**1.32**
	2	79	133	94	186	1.38	1.93
10^7^ CFU/mL inoculum	0.5	75	84	75	83	0.87	0.86
	1	85	**134**	84	**133**	**1.40**	**1.39**
	2	92	137	94	195	1.43	2.03

*^*a*^*Figures for f*AUC_*PK*(*0*-*96*__*h*)_/MIC (h) *were computed from equation 8 for two inoculum strengths* (*10^*5*^ and 10^*7*^ CFU/mL*) *and for MICs of 0.5, 1, and 2 mg/L.*^*b*^*The corresponding average plasma concentration to achieve over 96h* (*expressed in multiples of MIC*) *to ensure 90% efficacy was computed by dividing these critical values by 96h*. Bold fonts highlight values for MIC of 1 mg/L.*

Data in Table 2 indicate that the critical value of the PK/PD index (*f*AUC_PK(0-96 h)_/MIC) to achieve 90% of maximal effect) was dependent of the tested MIC but relatively similar for both bacterial species. For a MIC of 1 mg/L, the critical value for *f*AUC_PK(0-96 h)_/MIC for *P. multocida* was 115 and 134 h for inocula of 10^5^ and 10^7^ CFU/ml, respectively. The corresponding values were 127 and 133 h for *M. haemolytica*. These values indicate that, to achieve 90% of maximal efficacy for a pathogen having a MIC of 1 mg/L, the average free plasma florfenicol concentration over 96 h should be equal to 1.19- and 1.32-fold the MIC for *P. multocida* and 1.40 and 1.39 fold the MIC for *M. haemolytica*.

## Discussion

This study is the first to quantify, for veterinary pathogens, the three basic PD parameters of an AMD from TKC analysis, namely efficacy (E_MAX_ maximum killing rate), potency (EC_50_) and sensitivity (slope of the concentration-effect relationship). These data have been obtained for florfenicol and two major calf pathogens, *P. multocida* and *M. haemolytica*. The classical index describing quantitatively AMD action is MIC. However, MIC is not a genuine PD parameter; it is a reproducible hybrid variable measured under standard conditions. Actually, MIC is dependent not only on the three PD parameters but also on *in vitro* conditions (growth and death rates of the tested pathogen, duration of observation and the initial inoculum load). The numerical value of each MIC therefore depends on seven separate factors, as explicitly indicated in eqs. 6A,B.

The advantage of dissecting MIC into these dependency components is to identify the test tube conditions that can be regarded as confounding factors from the actual PD properties that are of primary interest, not only for AMDs but for drugs of all classes. The three parameters have been dissected out and quantified by modeling TKC data. In contrast with MIC, as a crude index of AMD action, TKCs describe time course as well as magnitude of antibacterial action over the 18–24 h duration of exposure. This enables capture of the pattern of bacterial killing with semi-mechanistic models of the type used in the present paper (Figure 1). This model has recently been evaluated against similar PK/PD models proposed by others and using Monte Carlo Simulations. It was concluded that, under constant drug concentrations, as in this study, the median PD parameter estimates were within 10% of the true value and the precision was < 20% (Jacobs et al., 2016).

The calculations indicate that potency and efficacy of florfenicol were of the same order of magnitude for the two pathogens investigated. The strains belonged to the distribution of the wild population for the two pathogens as EUCAST epidemiological cut-off values (ECOFFs) values are 1 and 2 mg/L for *P. multocida* and *M. haemolytica*, respectively. In future studies, it would be valuable to subject to the same modeling process strains belonging to resistant sub-populations; this would reveal how resistance is phenotypically expressed (for example, as either an increase in EC_50_ and/or a reduction in E_MAX_). Such data would enable interpretation of mechanisms of emergence of resistance, using the same conceptual framework for drugs of other pharmacological classes, when analyzing drug-receptor interaction. Such analysis is a major tool in the quest for developing new drugs (Kenakin, 1997).

The analysis presented in this paper adds a new dimension to bactericidal killing curves by converting them into proxies of an infection model. This required linking *in vivo* PK data to a PD TKC model able to predict the temporal dynamics of bactericidal activity. The PK data are generated by solving a model readily obtained through either classical or population investigations. The PD model predicts a microbiological response for a given drug exposure at two inoculum levels of 10^5^ and 10^7^ CFU/mL (corresponding to metaphylactic and treatment circumstances, respectively). Currently, rodent models are widely used but they raise questions of cost and ethical use of animals in research. As an alternative to animal studies, the hollow fiber model has been developed as a dynamic infection model (Michael et al., 2014) but its use in veterinary medicine has not yet been reported. Hollow fiber technology is costly and resource demanding; few alternatives like chemostats can be explored but have their own limitations. The present adaptation of TKC results offers the advantages of using historical data and its availability for many veterinary pathogens. Hence, data meta-analysis, as presented in this article, provides, at low cost and with benefits for animal ethics, a new approach to selection of a PK/PD index to predict clinical efficacy of AMDs used in veterinary medicine.

The selected PD model simulated the time course of bactericidal activity of florfenicol, with pathogen exposure actually obtained *in vivo* after administration to calves of the reference florfenicol formulation (*Nuflor^®^*). To achieve this, the PD component of the model with its estimated parameters was solved using plasma florfenicol concentrations as predicted by a florfenicol population model (Supplementary File S3, also see Toutain et al., 2019).

Thus, several florfenicol exposure scenarios were simulated to generate corresponding killing curves. This leads to the conclusion that a single florfenicol dose of 40 mg/kg should be more efficacious in bactericidal effect than an alternative dosing regimen of two 20 mg/kg dose at a 48 h interval. According to a meta-analysis from DeDonder and Apley (2015), both dosage regimen were equally efficacious (absolute risk reduction of morbidity) versus negative control.

In order to propose a PK/PD breakpoint for florfenicol based on the VetCAST approach, the first step is to select an appropriate PK/PD index predicting efficacy. A PK/PD approach is superior to using a target CFU at 24 h as it allows the description of the onset, rate and extent of killing and a data-based determination as to whether an AMD is time or concentration-dependent. Florfenicol is used solely in veterinary medicine, so that historically no dose fractionation rodent studies are available to determine the most appropriate PK/PD index predicting efficacy. For determination of the best PK/PD index, *in silico* simulation approaches are scientifically attractive, ethically acceptable and low cost alternatives to *in vivo* dose fractionation studies. This *in silico* approach has been validated for human medicine for the main AMD classes (Nielsen and Friberg, 2013). To select *f*AUC/MIC or *f*T > MIC as the PK/PD index of choice, it is necessary to establish the influence of both level (concentration) and shape of exposure to florfenicol on the efficacy of its bactericidal effect, as predicted by the PD model. In this study, from simulated killing curves obtained with 10 florfenicol dose levels ranging from 0 to 80 mg/kg and divided into one, two or four administrations at differing dosing intervals, 28 killing curve profiles were generated. These were then modeled using the classical E_max_ model, with the PK/PD index as independent variable and fAUC_(0-96 h)_ under the killing curves as dependent variable. For MICs of 0.5, 1, and 2 mg/L, *f*AUC/MIC was systematically superior to *f*T > MIC in predicting bacterial killing, although for the lowest MIC (0.5 mg/L) both indices were acceptable. For an MIC of 2 mg/L, the relationship degraded for *f*T > MIC but remained acceptable for *f*AUC/MIC. For an MIC of 4 mg/L, both indices failed to predict adequately the florfenicol response or lack thereof.

The selection of *f*AUC/MIC as the best PK/PD index for florfenicol is consistent with a previous report that, regardless of AMD class, *f*AUC/MIC is the most appropriate index when terminal half-life is long (Nielsen and Friberg, 2013).

In this study, results of simulations are presented using free plasma concentration of florfenicol, as free plasma concentration is the best proxy for concentration in the biophase. In non-lactating dairy cattle, plasma protein binding ranged from 19 to 23% (Bretzlaff et al., 1987). However, a recent investigation reported that the degree of florfenicol binding in 6-month old steers was either very low (5%) or negligible (Foster et al., 2016). Such low binding differs from another recent study (Mzyk et al., 2018). Investigating the influence of age (1 to 168 days) on degree of florfenicol plasma protein binding, these authors reported binding ranging from 12 to 42% in one-day old, and from 11 to 32% in 168-day old animals, at a concentration of 1 mg/L. In light of these inter-study differences, and as the selected PK/PD index is *f*AUC/MIC, it would be a simple matter to apply a correction for unbound fraction during the computation of the PK/PD cut-off for florfenicol by Monte Carlo simulation. On this basis, it is concluded that for both *P. multocida* and *M. haemolytica* maximum efficacy (actually 90%) over 96 h is obtained when the average free plasma concentration is equal to the 1.2 to 1.4 times the respective MIC.

## Data Availability

All datasets analyzed for this study are included in the manuscript and the Supplementary Files (S1–S2 ).

## Author Contributions

PL and PS generated raw data. LP retrieved and validated raw data. P-LT and LP performed the modeling analysis and drafted the manuscript. All authors critically reviewed several drafts of the manuscript.

## Conflict of Interest Statement

The authors declare that the research was conducted in the absence of any commercial or financial relationships that could be construed as a potential conflict of interest.
