# Supplementary material for: Semi-Mechanistic Modeling of Florfenicol Time-Kill Curves and in silico Dose Fractionation for Calf Respiratory Pathogens
Source: Front Microbiol. 2019 Jun 11;10:1237. doi: 10.3389/fmicb.2019.01237 (PMC6579883; doi:10.3389/fmicb.2019.01237)
Supplement: Supplementary file 1 [file Table_1.DOCX]

***Supplementary file S1: origin of the bacterial strains***

*M. haemolytica* and *P. multocida* strains were supplied on swabs by the Veterinary Laboratory Agency (VLA). All organisms were derived from field cases of calf pneumonia between 2004 and 2007 in different geographical regions of the United Kingdom (**Table 1-S1**). Twenty strains of each species were tested for their ability to multiply logarithmically in Mueller Hinton Broth and 6 strains of each selected for use in this study. This pre-selection ensured that all strains could be studied in MIC, MBC and time-kill investigations.

**Table 1-S1**. Origin of the bacterial strains selected for use in experiments to determine pharmacodynamic properties of florfenicol against *M. haemolytica* and *P. multocida*.

| **Organism** | **Reference** | **Age of calf (months)** | **VLA centre** | **Submission Date** |
| --- | --- | --- | --- | --- |
| *M. haemolytica* | 1056 | 3 | Shrewsbury | 26/06/2004 |
| *M. haemolytica* | 1250 | 4 | Aberystwyth | 29/11/2004 |
| *M. haemolytica* | 1978 | >6 | Preston | 17/10/2005 |
| *M. haemolytica* | 2008 | 10 | Bury St Edmunds | 14/10/2005 |
| *M. haemolytica* | 2059 | >6 | Bristol | 23/11/2005 |
| *M. haemolytica* | 2653 | 6 | Bristol | 23/05/2006 |
| *P. multocida* | 3722 | 8 | Truro | 16/11/2006 |
| *P. multocida* | 3979 | 3.5 | Bristol | 23/01/2007 |
| *P. multocida* | 4072 | 6 | Preston | 17/01/2007 |
| *P. multocida* | 4096 | 3 | Winchester | 19/01/2007 |
| *P. multocida* | 4121 | >6 | Preston | 17/02/2007 |
| *P. multocida* | 4323 | 4 | Newcastle | 13/03/2007 |
