# Supplementary material for: Semi-Mechanistic Modeling of Florfenicol Time-Kill Curves and in silico Dose Fractionation for Calf Respiratory Pathogens
Source: Front Microbiol. 2019 Jun 11;10:1237. doi: 10.3389/fmicb.2019.01237 (PMC6579883; doi:10.3389/fmicb.2019.01237)
Supplement: Supplementary file 3 [file Table_3.docx]

Supplemental file S3: population pharmacokinetic model

This file summarises the construction of the population pharmacokinetic model used for the simulation part of the present manuscript. The paper reporting the model has been submitted for publication (*VetCAST method for determination of the pharmacokinetic-pharmacodynamic cut-off values of florfenicol to support clinical breakpoints for florfenicol Antimicrobial Susceptibility Testing in cattle*, P-L Toutain, P.K. Sidhu, P. Lees 2, A. Rassouli and L. Pelligand, in press in Frontiers in Microbiology, DOI: 10.3389/fmicb.2019.01310)

# Source of the plasma concentration-time curves

Individual calf PK data from three different sources (A=10, B=32 C=8) were used for the POP PK analysis. Source A consisted of 10 calves from a published study (Sidhu et al., 2014). Source B was a drug company (Norbrook Laboratories Limited); it comprised 16 calves enrolled in a cross-over bioequivalence study (MSD Nuflor® and Norbrook Norfenicol® formulations); Norfenicol® being a FDA and EMA approved generic product (Anonymous, 2018a). The 32 data sets were provided by 16 sets for each product, so that for this analysis each of these calves provided two data sets. The third source comprised data from 8 calves in an unpublished study (Lees et al). All calves were in good health and all received a subcutaneous florfenicol dose of 40 mg/kg.

# Population pharmacokinetic model

Pharmacokinetic data analyses were carried out using Phoenix® WinNonlin® 8.0 (Pharsight Corporation St Louis, MO, USA). Data sets obtained from the three sources were analyzed using a Non-Linear Mixed Effect model (NLME). A two-compartmental model (macro constant parametrization) was selected based on the Likelihood Ratio Test (LRT), the Akaike Information Criterion (AIC) and inspection of different diagnostic plots.

The parametrization of the structural two-compartmental model was of the closed form (Equation 1):

$\boldsymbol{C}\left( \boldsymbol{t} \right)\mathbf{=}\boldsymbol{A}\boldsymbol{\times}\boldsymbol{EXP}\left( \mathbf{-}\boldsymbol{Alpha}\boldsymbol{\times}\boldsymbol{t} \right)\mathbf{+}\boldsymbol{B}\boldsymbol{\times}\boldsymbol{EXP}\left( \mathbf{-}\boldsymbol{Beta}\boldsymbol{\times}\boldsymbol{t} \right)\mathbf{-(}\boldsymbol{A}\mathbf{+}\boldsymbol{B}\boldsymbol{)\times}\boldsymbol{EXP}\mathbf{(-}\boldsymbol{Ka}\boldsymbol{\times}\boldsymbol{t}\mathbf{)}$ Eq:1

where macroconstants *t* is the time (h), *A* and *B* (µg/ml) are intercepts and *Alpha*, *Beta* and *Ka* are rate constants (1/h) associated with the phases of plasma concentration-time profile. Parametrization was in terms of macroconstants and rate constants rather than in terms of clearance and volume of distribution for reasons explained in the Discussion. The five fixed parameters (described as vector Thetas) were estimated and reported as typical values (tv) with coefficient of variation as a measure of precision of the estimate (Supplementary Table 1). The random component that describes biological variability around the structural fixed parameters i.e. the Between-Subject Variability (BSV) across individuals was described by an exponential model of the form (Equation 2):

$\boldsymbol{\theta}_{\mathbf{1}\boldsymbol{i}}\mathbf{=}\boldsymbol{\theta}_{\mathbf{1}}\boldsymbol{\times}\boldsymbol{Exp}\mathbf{(}\boldsymbol{\eta}_{\mathbf{1}\boldsymbol{i}}\mathbf{)}$ Eq: 2

where $\theta_{1}$ is the typical population value of theta (*A*, *B*, *Alpha*, *Beta* or *Ka*) , $\theta_{1i}$ the value of theta in the i^th^ animal, and $\eta_{1i}$ (eta) the deviation associated with the i^th^ animal from the corresponding theta population value. This exponential model assumes a log-normal distribution of parameters, i.e. that the distribution of the etas is normal in the log-domain, with a mean of 0 and a variance ω^2^ where:

$$\boldsymbol{\eta}\boldsymbol{\approx}\boldsymbol{N}\mathbf{(0,}\boldsymbol{\omega}^{\mathbf{2}}\mathbf{)}$$

Each eta distribution associated to each theta with its own variance $\omega_{A}^{2}$ , $\omega_{Alpha}^{2} \omega_{B}^{2}$, $\omega_{Beta}^{2}$or $\omega_{Ka}^{2}$was computed, but covariance terms between etas have been ignored (diagonal matrix).

The BSV was reported as coefficient of variation in the original scale with the following equation that converts the variance terms ($\omega^{2}$) to a coefficient of variation (CV%).

$\mathbf{CV}\left( \boldsymbol{\%} \right)\boldsymbol{=100\times}\sqrt{\exp\left( \boldsymbol{\omega}^{\mathbf{2}} \right)\mathbf{-1}}$ Eq:3

The residual variability was modeled with an additive and a multiplicative component. Like other random-effects, the residual error can be dependent on subject-specific covariate of the analytical technique used to generate plasma concentration (Bonate, 2011). In the error model a covariate was included to take account of the fact that three differing analytical techniques were used to generate the data set.

The residual error model without covariate was of the form (equation 4):

$\mathbf{Y=}\boldsymbol{f}\left( \boldsymbol{\theta}\mathbf{,}\boldsymbol{Time} \right)\boldsymbol{\times}\left( \mathbf{1+}\boldsymbol{\varepsilon}_{\mathbf{1}} \right)\mathbf{+}\boldsymbol{\varepsilon}_{\mathbf{2}}$ Eq.4

with ε1 the multiplicative error term having a mean of 0 and a variance of σ1

$$\boldsymbol{\varepsilon}\boldsymbol{1\approx}\boldsymbol{N}\mathbf{(0,}{\boldsymbol{\sigma}\mathbf{1}}^{\mathbf{2}}\mathbf{)}$$

and ε2 the common additive error term having a mean of 0 and a variance noted σ2

$$\boldsymbol{\varepsilon}\boldsymbol{2\approx}\boldsymbol{N}\mathbf{(0,}{\boldsymbol{\sigma}\mathbf{2}}^{\mathbf{2}}\mathbf{)}$$

Sigma1 and Sigma2 were estimated by Phoenix and reported as a CV% for sigma1 and as a STDV for sigma2.

Secondary parameters were also computed (terminal half-lives for the first and second phase of drug disposition and contribution of the first and second phases to drug absorption) and reported in Supplementary Table 1.

**Supplementary Table 1-S3**: Population primary (Thetas) and secondary parameters and random effects (Omega) for florfenicol in 50 calves obtained with a 2-compartment model. Florfenicol was administered at a 40mg/kg dose, subcutaneously.

| THETAS | **Estimate** | **Units** | **SE** | **CV%** | | **2.5% CI** | | **97.5% CI** |
| --- | --- | --- | --- | --- | --- | --- | --- | --- |
| tvKa | 0.975 | 1/h | 0.123 | 12.66 | | 0.733 | | 1.218 |
| tvA | 5.05 | µg/ml | 0.2368 | 4.69 | | 4.59 | | 5.52 |
| tvAlpha | 0.0442 | 1/h | 0.0041 | 9.35 | | 0.0361 | | 0.0523 |
| tvB | 0.781 | µg/ml | 0.243 | 31.13 | | 0.304 | | 1.258 |
| tvBeta | 0.0104 | 1/h | 0.0019 | 18.31 | | 0.0067 | | 0.0141 |
| tvC1MultStdev | 0.1397 |  | 0.014 | 10.37 | | 0.111 | | 0.168 |
| tvC1MultStdev | 13.970 | % |  |  | |  | |  |
| stdev0 | 0.0152 | µg/ml | 0.0112 | 73.64 | | -0.0068 | | 0.0371 |
| OMEGA | **Variance** | **SE** | **BSV (CV%)** |  |  | |  |  |
| nKa | 0.279 | 0.076 | 56.69 |  |  | |  |  |
| nAlpha | 0.033 | 0.012 | 18.23 |  |  | |  |  |
| nB | 0.036 | 0.051 | 19.11 |  |  | |  |  |
| nBeta | 0.103 | 0.040 | 32.97 |  |  | |  |  |
| nA | 0.080 | 0.024 | 28.92 |  |  | |  |  |
| Secondary parameters | **Estimate** | **Units** | **SE** | **CV%** | | **2.5% CI** | | **97.5% CI** |
| Half-life Alpha | 15.7 | h | 1.46 | 9.35 | | 12.8 | | 18.5 |
| Half-life Beta (t1/2) | 66.7 | h | 12.21 | 18.31 | | 42.7 | | 90.6 |
| AUC (0-infinity) | 183.4 | µg*h/ml | 3.41 | 1.86 | | 176.1 | | 190.1 |
| Absorption first phase | 0.603 | Fraction | 0.058 | 9.64 | | 0.489 | | 0.717 |
| Absorption second phase | 0.397 | Fraction | 0.058 | 14.66 | | 0.283 | | 0.511 |

*For interpretation of parameters, see equations 1 (Thetas) and 2 (Omega). AUC was obtained by integrating equation1 with estimated tv of thetas parameters. The disposition of florfenicol for the investigated formulations obeys a flip-flop pattern (see Discussion) and fraction absorbed during the first versus the second phase was estimated by computing partial areas associated with the alpha phase (A/Alpha) and the beta phase (B/Beta).*

**Reference of the supplementary materials**

Anonymous (2018a). NORFENICOL- florfenicol injection, solution Norbrook Laboratories Limited. Available at: <https://www.norbrook.com/products/united-states/norfenicol-injectable-solution-florfenicol> [Accessed August 22, 2018].
